# Supplementary material for: Structural consequences of turnover-induced homocitrate loss in nitrogenase
Source: Nat Commun. 2023 Feb 25;14:1091. doi: 10.1038/s41467-023-36636-4 (PMC9968304; doi:10.1038/s41467-023-36636-4)
Supplement: Supplementary file 1 — Supplementary Information [file 41467_2023_36636_MOESM1_ESM.pdf]

Supplementary Table 1: Cryo-EM data collection, refinement, and validation statistics

**Datasets**	**MoFe**$^{\text{As-isolated}}$	**MoFe**^{Oxidized}	**MoFe**^{Alkaline}	**MoFe**$^{\text{Alkaline-inactivated}}$	**MoFe**$^{\Delta\text{nifV}}$	**MoFe**$^{\Delta\text{nifV-NafT}}$
PDB IDs	8CRS	8DBX	8ENM	8ENL	8ENN	8ENO
Microscope	Titan Krios	Titan Krios	Titan Krios	Titan Krios	Titan Krios	Titan Krios
Camera	Gatan K3 Summit	Gatan K3 Summit	Gatan K3 Summit	Gatan K3 Summit	Gatan K3 Summit	Gatan K3 Summit
Magnification	130,000x	130,000x	130,000x	130,000x	130,000x	130,000x
Voltage (kV)	300	300	300	300	300	300
Recording mode	counting	counting	counting	counting	counting	counting
Frames/Movies	40	40	40	40	40	40
Total Electron dose (e-/Å2)	60	60	60	60	60	60
Defocus range (µm)	-0.8 to -3.0	-0.8 to -3.0	-0.8 to -3.0	-0.8 to -3.0	-0.8 to -3.0	-0.8 to -3.0
Pixel size (Å)	0.65	0.65	0.65	0.65	0.65	0.65
Micrographs collected	6,777	8,268	12,564	11,663	5,562	16,547
Total extracted particles	5,724,570	6,367,415	11,018,717	3,658,912	3,738,519	6,724,011
Refined particles	137,629	219,537	331,604	156,311	92,879	99,332
Symmetry imposed	C2	C2	C2	C1	C1	C1
Nominal Map Resolution (Å)	2.13	1.92	2.14	2.37	2.58	2.71
FSC threshold	0.143	0.143	0.143	0.143	0.143	0.143
masked/unmasked	1.9/2.0	1.8/1.9	1.9/2.0	2.1/2.1	2.2/2.4	2.5/2.6
Refinement						
Initial model used	3U7Q	3U7Q	3U7Q	3U7Q	3U7Q	3U7Q
Number of atoms						
Protein	17,884	16,986	17,722	16,242	16,749	16,485
Ligand	ICS:2; CLF: 2; HCA:2; FE:2; 1N7:4	ICS:2; CLF: 2; 1CL:2; HCA:2; FE:2	ICS:2; CLF: 2; HCA:2; FE:2;	ICS:2; CLF: 2; HCA:2; FE:2; 1N7:2	ICS:2; CLF: 2; HCA:2; FE:2; 1N7:2	ICS:2; CLF: 2; HCA:2; FE:2; 1N7:2
MapCC (mask/box)	0.90/0.77	0.83/0.75	0.90/0.78	0.88/0.74	0.89/0.76	0.89/0.79
Map sharpening B-factor	40	30	40	50	40	50
R.m.s. deviations						
Bond lengths (Å)	0.003	0.003	0.003	0.003	0.004	0.003
Bond angles (º)	0.631	0.566	0.545	0.524	0.552	0.537
MolProbity score	1.49	1.58	1.25	1.51	1.42	1.55
Clashscore (all atom)	9.26	9.2	4.77	5.48	6.65	6.92
Rotamer outliers (%)	0.99	0.81	0.75	1.53	0.94	1.53
Ramachandran plot						
Favored (%)	98.19	97.54	98.14	97.67	97.75	97.88
Allowed (%)	1.81	1.86	1.86	2.33	2.25	2.12
Outliers (%)	0.00	0.00	0.00	0.00	0.00	0.00

Supplementary Table 2: Summary of notable features in nitrogenase MoFe-protein cryoEM structures presented in this work.

**Feature**	**Dimer**	**$MoFe^{As\text{-}isolated}$**	**$MoFe^{Alkaline}$**	**$MoFe^{Alkaline\text{-}inactivated}$**	**$MoFe^{\Delta nifV}$**	**$MoFe^{\Delta nifV}$-NafT**
**Subunit Disorder**	Ordered	0	0	0	0	α36- α40 (5)
	Disordered	0	0	α1- α48; α354- α360; α376- α416; α423- α425 (90 residues)	α14- α19; α25- α26; α36- α40; α408 - α417 (23 residues)	α1- α48; α376 - α383; α390 - α398; α402 - α409; ε41- ε48 (82 residues)
**HCA loss**	Ordered	No	No	**Yes**	**Yes; citrate partial occupancy**	**Yes; citrate partial occupancy**
	Disordered	No	No	**Yes**	**Yes**	**Yes**
**Phe300 flip**	Ordered	No	No	**Yes**	No	**Yes**
	Disordered	No	No	**Yes**	**Yes**	**Yes**
**His274 flip**	Ordered	No	No	**Yes**	No	**Yes**
	Disordered	No	No	**Yes**	**Yes**	**Yes**
**His362 flip**	Ordered	No	No	No	No	No
	Disordered	No	No	**Yes**	No	**Yes**
**His451 flip**	Ordered	No	No	**Yes**	No	**Yes**
	Disordered	No	No	**Yes**	**Yes**	**Yes**
**His442 rearrangement**	Ordered	No	No	No	No	No
	Disordered	No	No	**Yes**	**Yes**	No
**His-triad/quartet**	Ordered	No	No	No	No	No
	Disordered	No	No	**Yes; quartet**	No	**Yes; triad**
**Trp253 flip**	Ordered	No	No	**Yes**	**Yes**	**Yes**
	Disordered	No	No	**Yes**	No	**Yes**
**Gln93 flip**	Ordered	No	No	No	No	No
	Disordered	No	No	**Yes**	No	No

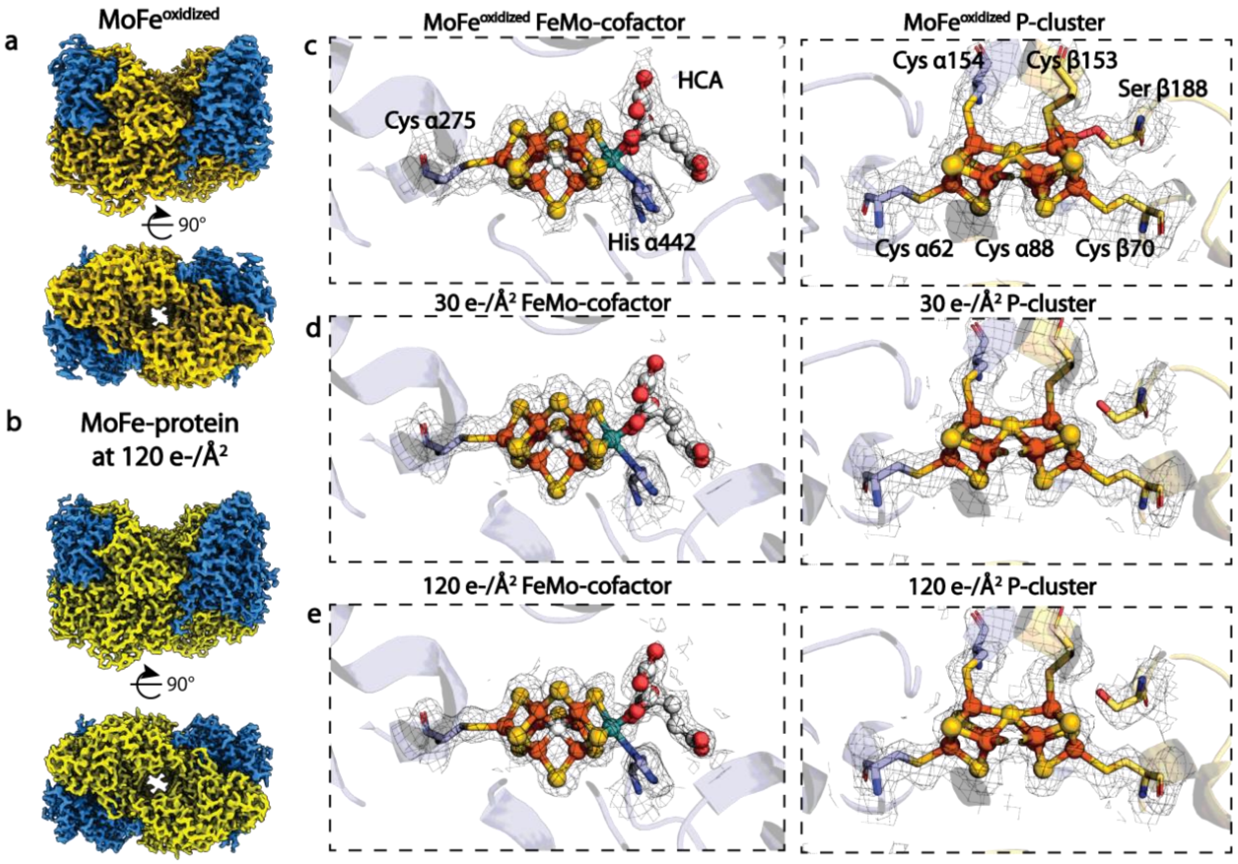

Supplementary Figure 1: Cryo-EM density of MoFe-protein upon exposure to oxygen or variable dose. a, 1.92 Å resolution cryo-EM map of $MoFe^{Oxidized}$. A sample of the MoFe-protein alone was transferred from a sealed anaerobic vial onto a grid within a benchtop Vitrobot (exposed to air) using a gastight syringe and immediately blotted and plunge frozen (<10 seconds). **b,** To probe the possible photoreductive effects of the electron beam on the metalloclusters present in the MoFe-protein, we subjected the anaerobically prepared sample to a dose of 120 e-/Å^2, panel b represents the resulting map. **c,** Analysis of the $MoFe^{oxidized}$ map surrounding the P-cluster revealed bridging density between Fe6 and Ser β188, but no bridging density between Fe5 and the backbone amide of Cys α88. Consequently, the aerobically frozen MoFe-protein cryo-EM structure corresponds to the oxidized P^{+1} state of the P-cluster, emphasizing the necessity for anaerobic cryo-EM sample preparation conditions for the study of MoFe-nitrogenase **d**, Density around the metalloclusters in the low dose (30 e-/Å^2) map. **e,** Density around the metalloclusters in the high dose (120 e-/Å^2) map. Abbreviations: e-/Å^2, electrons per Ångstroms squared; HCA, *R*-homocitrate.

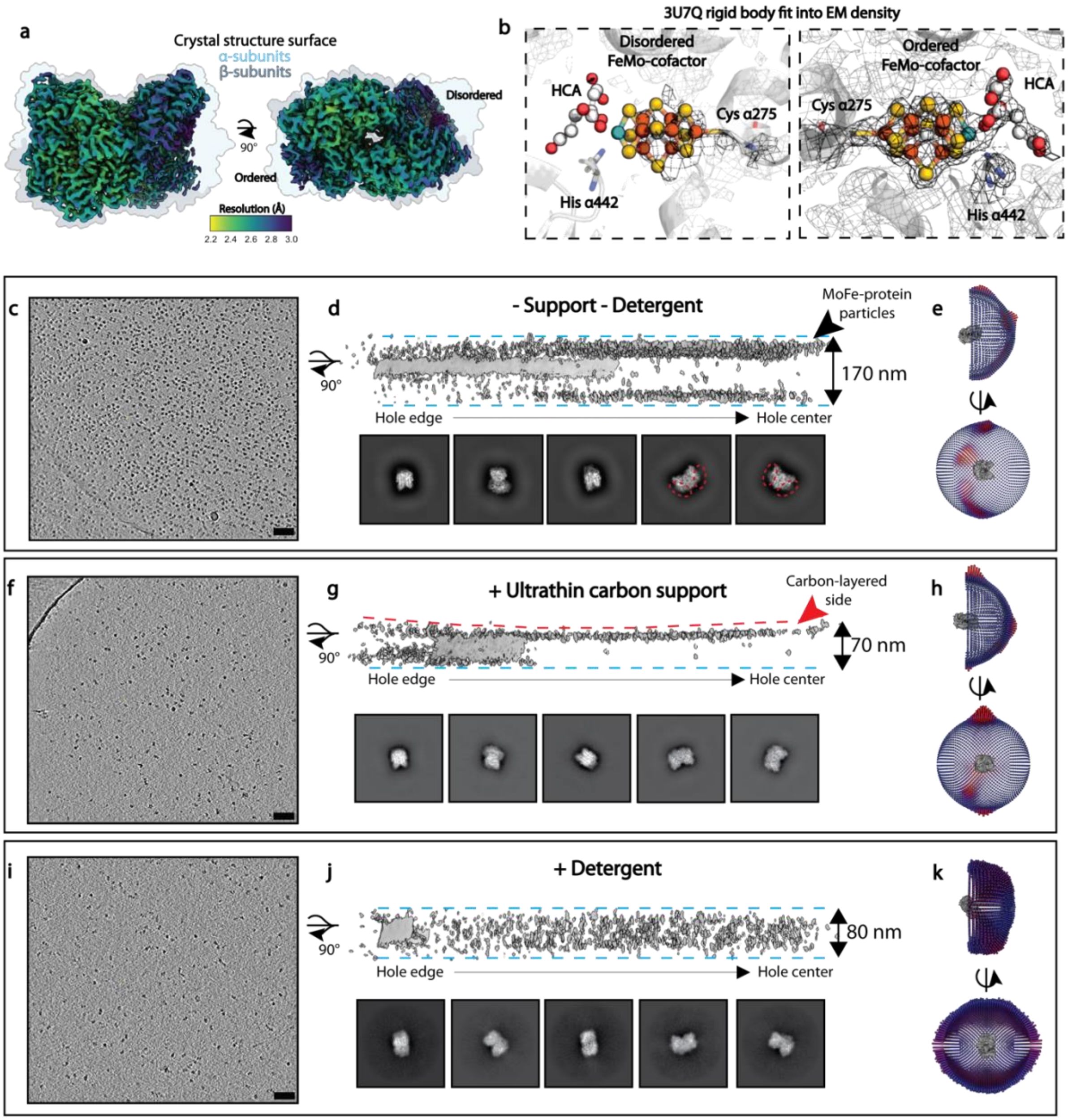

Supplementary Figure 2: Detergent supplementation limits air water interface adsorption and preferred orientation of MoFe-nitrogenase particles. a, 2.56 Å resolution (C1-symmetry) cryo-EM map of the MoFe-protein shaded according to local resolution overlaid with surface representation of MoFe-protein crystal structure (PDB code 3U7Q). **b,** Cryo-EM ESP at 7.5 σ of the metalloclusters overlaid with a rigid body fit of PDB code 3U7Q. **c-k,** Top panel: MoFe-protein particles alone on holey carbon grids. Middle panel: MoFe-protein particles alone on ultrathin carbon layered grids. Bottom panel: MoFe-protein particles with detergent on holey carbon grids. (**c, f, i**) reconstructed tomogram of MoFe-protein particles. Scale bar represents 100 nm (**d, g, j**) Top: Volumetric representation of tomogram showing particle distribution in ice. The solid gray features correspond to the reconstructed volumes of the hole edge. Bottom: Representative 2D classes from corresponding single particle reconstructions. (**e, h, k**) Euler angle distributions from reconstructed single particle cryo-EM maps (Red, overrepresented views). Abbreviations: Å, Ångstroms; HCA, *R*-homocitrate; nm, nanometers.

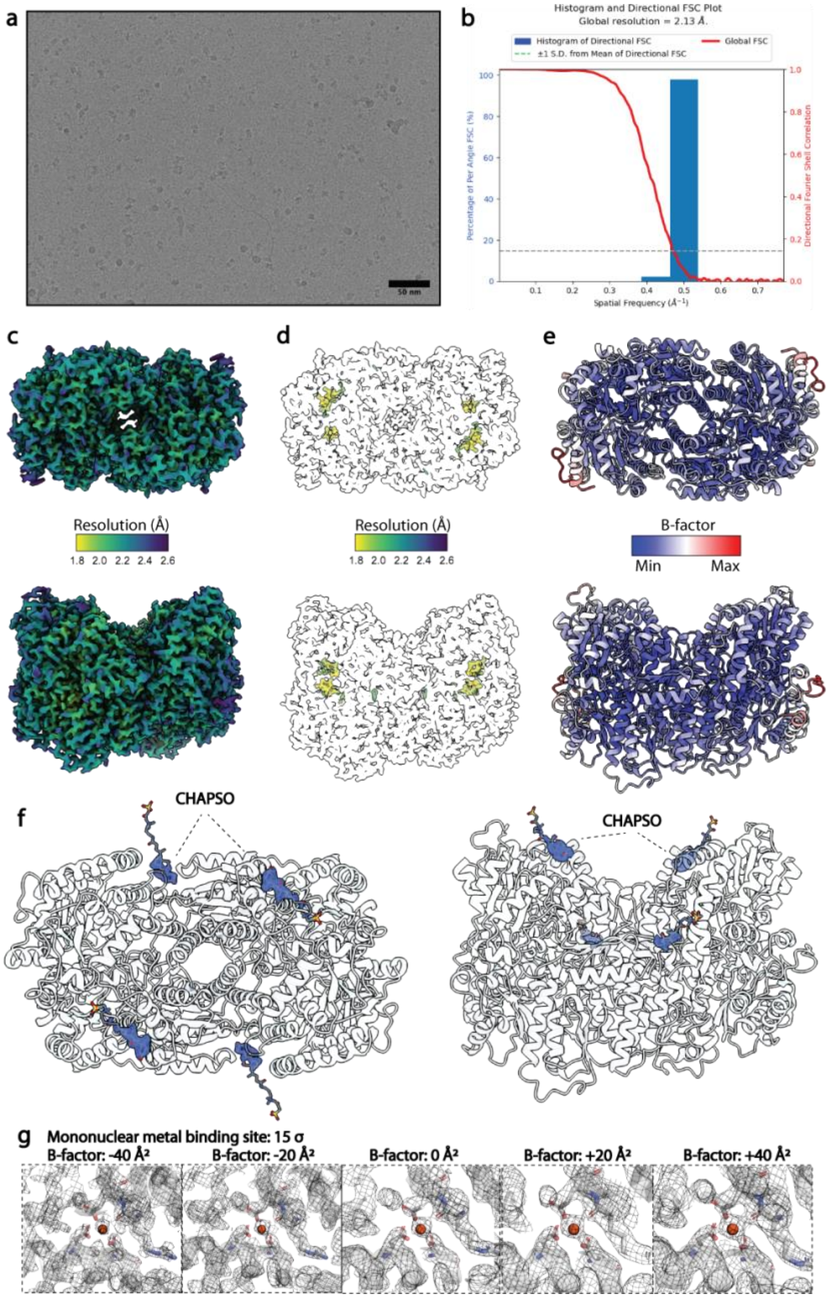

Supplementary Figure 3: Anaerobic, as-isolated MoFe-protein reconstruction with detergent. a, Representative micrograph of MoFe-protein particles on holey carbon grids with CHAPSO from a dataset of 6,777 micrographs. Scale bar corresponds to 50 nm**. b,** Half map FSC curve for reconstructed volume with C2 symmetry imposed. Curve correlates to FSC calculated with a tight mask. **c,** Cryo-EM map color-coded according to local resolution estimates. **d,** Cryo-EM map color-coded according to local resolution estimates at high threshold showing local resolution at metallocluster sites. **e,** Model color-coded according to B-factor. **f,** Electrostatic potential (ESP) map for bound CHAPSO molecules. **g,** Cryo-EM ESP for the previously identified mononuclear metal binding site modeled as Fe with the map sharpened or blurred at indicated B-factors. This site is distinct from other coordination sites identified within this study, such as that shown in Figures 3, 4, and Supplementary Figure 8. Abbreviations: Å, Ångstroms; HCA, *R*-homocitrate; nm, nanometers; FSC, Fourier Shell Correlation; CHAPSO, 3-([3-Cholamidopropyl]dimethylammonio)-2-hydroxy-1-propanesulfonate.

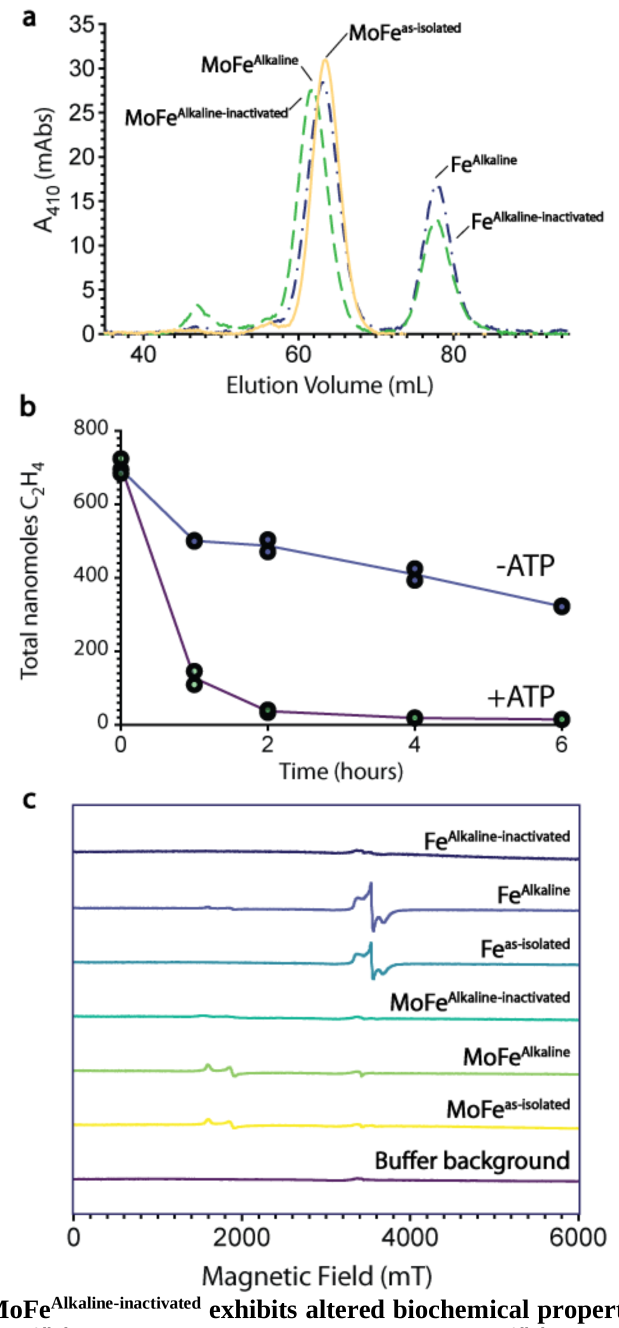

Supplementary Figure 4: $MoFe^{Alkaline\text{-}inactivated}$ exhibits altered biochemical properties. a, Separation of $MoFe^{As\text{-}isolated}$ (solid line, yellow), $MoFe^{Alkaline}$ (dashed-dotted line, green), and $MoFe^{Alkaline\text{-}inactivated}$ (dashed line, blue) by size exclusion chromatography monitoring the elution of the metalloproteins by absorbance at 410 nm. **b,** Specific activity assay monitoring inhibition and inactivation of MoFe-protein at pH 9.5 overtime with (+ATP, green circles) or without (-ATP, blue circles) turnover (n=2 reactions per condition, technical replicates). **c,** Electron paramagnetic resonance spectra of the $MoFe^{Alkaline\text{-}inactivated}$ and Fe-protein isolated from the alkaline inactivation reactions ($Fe^{Alkaline\text{-}inactivated}$) and control proteins. Source data are provided as a Source Data file. Abbreviations: ATP, Adenosine triphosphate; $Fe^{As\text{-}isolated}$, purified Fe-protein; $Fe^{Alkaline}$, Fe-protein isolated from control, no turnover (-ATP) alkaline reactions; $Fe^{Alkaline\text{-}inactivated}$, Fe-protein isolated from alkaline turnover reactions; C_2H_4, ethylene; mAbs, milli-absorbance units; mT, milliTesla.

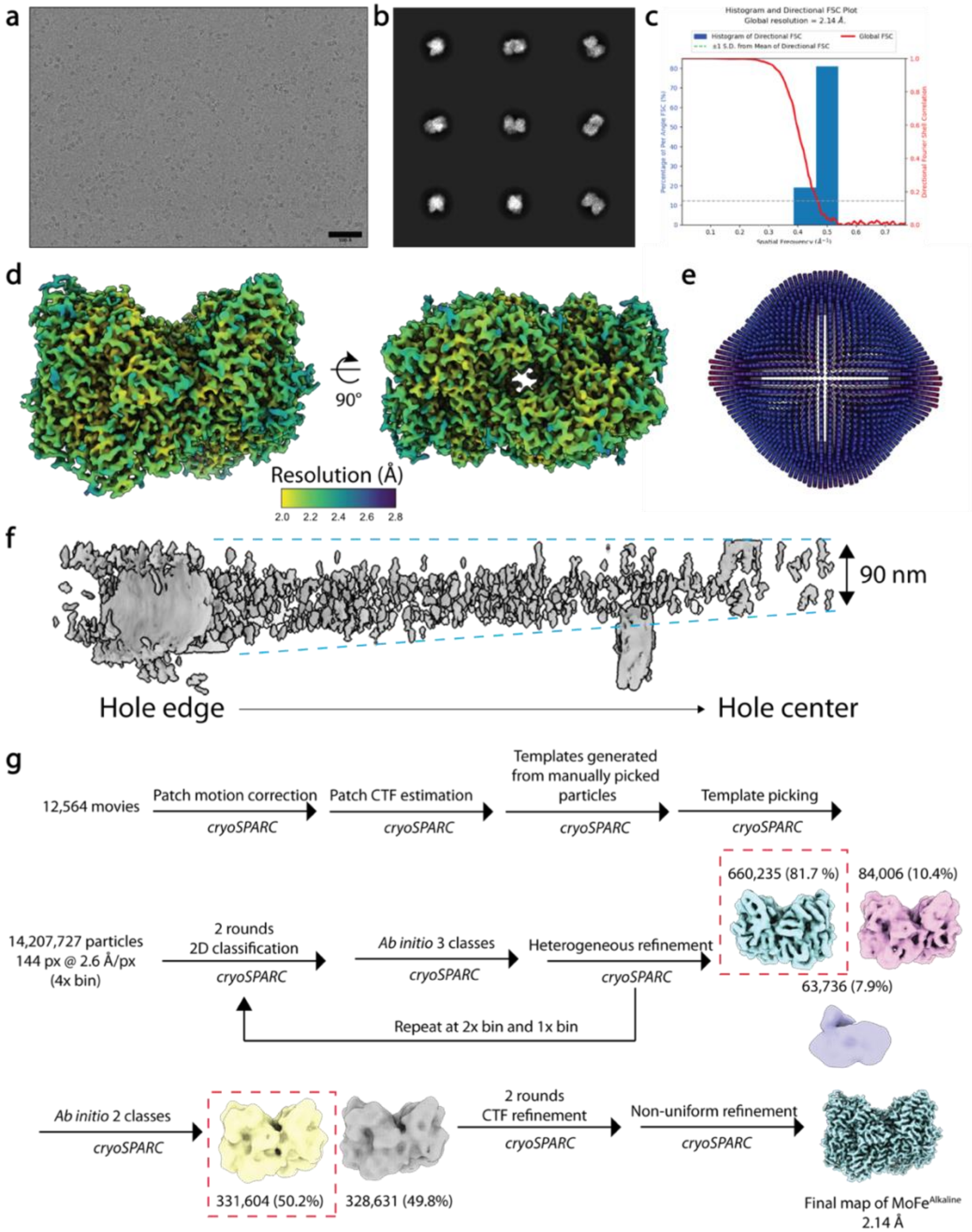

Supplementary Figure 5: Single particle cryoEM characterization of MoFe^{Alkaline} control structure. a, Representative micrograph of MoFe^{Alkaline} particles from a dataset of 12,564 micrographs. **b,** Representative 2D classes of MoFe^{Alkaline} particles **c,** Half map FSC curve for reconstructed volume. **d**, Cryo-EM map color-coded according to local resolution estimates. **e**, Euler angle distributions from reconstructed single particle cryo-EM map (Red, overrepresented views). **f,** Volumetric representation of tomogram showing particle distribution in ice. The solid gray features correspond to the reconstructed volumes of the hole edge. **g**, CryoEM data processing workflow. Abbreviations: Å, Ångstroms; nm, nanometers; FSC, Fourier Shell Correlation; px, pixel; CTF, contrast transfer function.

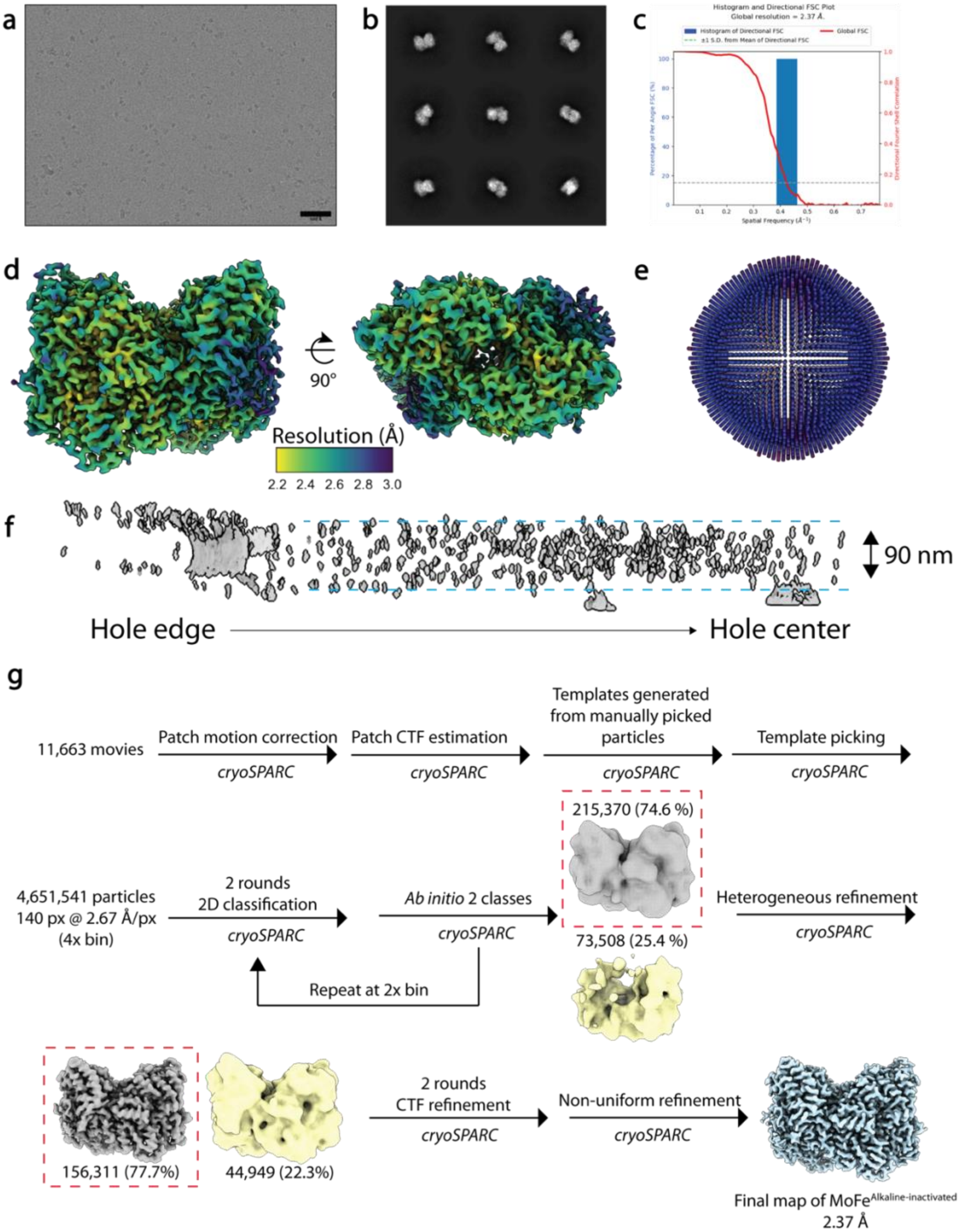

Supplementary Figure 6: Single particle cryoEM characterization of MoFe$^{\text{Alkaline-inactivated}}$. a, Representative micrograph of MoFe$^{\text{Alkaline-inactivated}}$ particles from a dataset of 11,663 micrographs. **b,** Representative 2D classes of MoFe$^{\text{Alkaline-inactivated}}$ particles **c,** Half map FSC curve for reconstructed volume. **d**, Cryo-EM map color-coded according to local resolution estimates. **e**, Euler angle distributions from reconstructed single particle cryo-EM map (Red, overrepresented views). **f,** Volumetric representation of tomogram showing particle distribution in ice. The solid gray features correspond to the reconstructed volumes of the hole edge. **g**, CryoEM data processing workflow. Abbreviations: Å, Ångstroms; nm, nanometers; FSC, Fourier Shell Correlation; px, pixel; CTF, contrast transfer function.

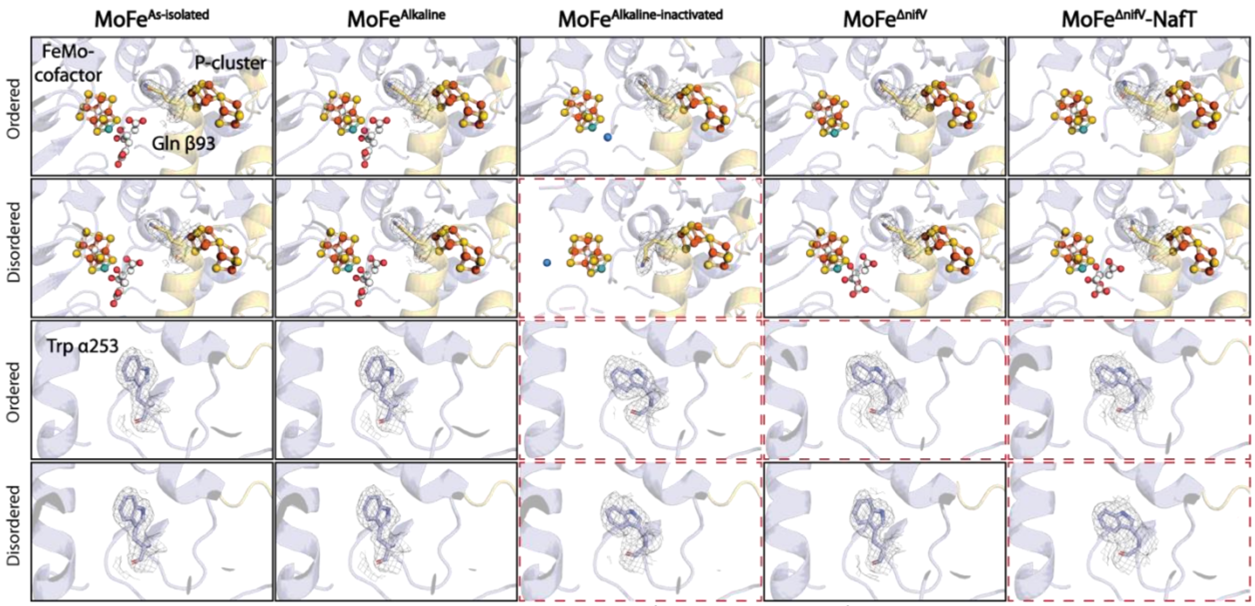

Supplementary Figure 7: MoFe$^{\text{Alkaline-inactivated}}$, MoFe$^{\Delta\text{NifV}}$, and MoFe$^{\Delta\text{NifV}}$-NafT structures display altered conformations of side chains Gln β93 and Trp α253. The Gln β93 and Trp α253 environments are shown in both the ordered and disordered subunits of all cryoEM structures presented in this work. Images boxed in dashed, red lines indicate residues that are changed with respect to the as-isolated state.

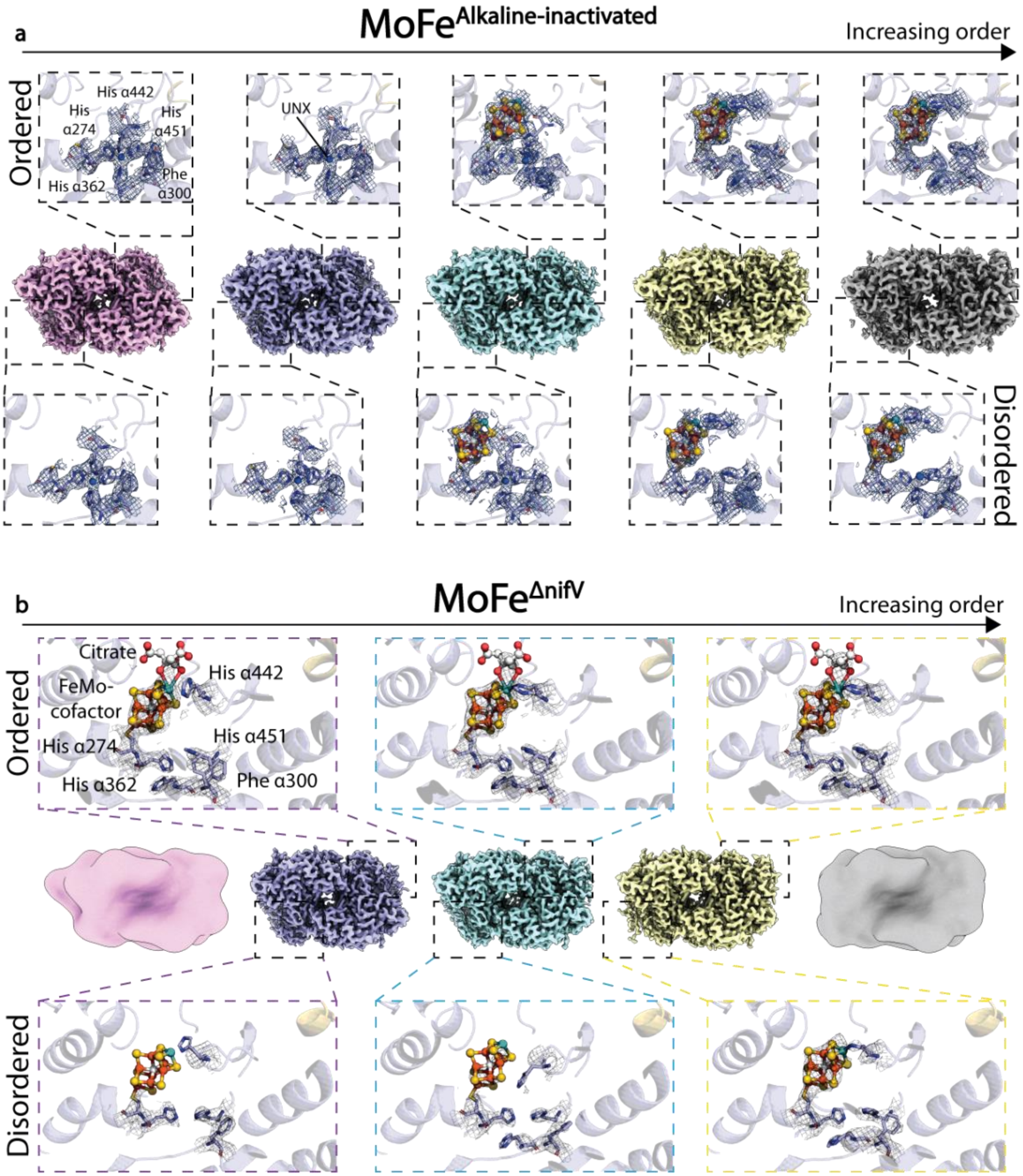

Supplementary Figure 8: Three dimensional variability analysis reveals varying states of order in the MoFe$^{Alkaline\text{-}inactivated}$ and MoFe$^{\Delta NifV}$ α-subunits. a, Five intermediate states were isolated by 3DVA in cryoSPARC, revealing increasingly ordered states as detected by the increasing polypeptide density in the α-subunit N-termini (boxed regions) and the reappearance of FeMo-cofactor density. Interestingly, in the two most ordered maps, the His-coordination site disappears as the Phe α300 side chain flips conformations. **b**, Five intermediate states were isolated by 3DVA in cryoSPARC, revealing increasingly ordered states as detected by the increasing polypeptide density in the α-subunit N-termini (boxed regions) and the reappearance of FeMo-cofactor density. While the first and last maps were too low resolution to model into, in the second map from left, it can be seen that Phe α300 in the more ordered subunit is in two conformations, suggesting that as the subunit becomes more disordered the side chain flips out, as seen consistently in the more disordered subunit.

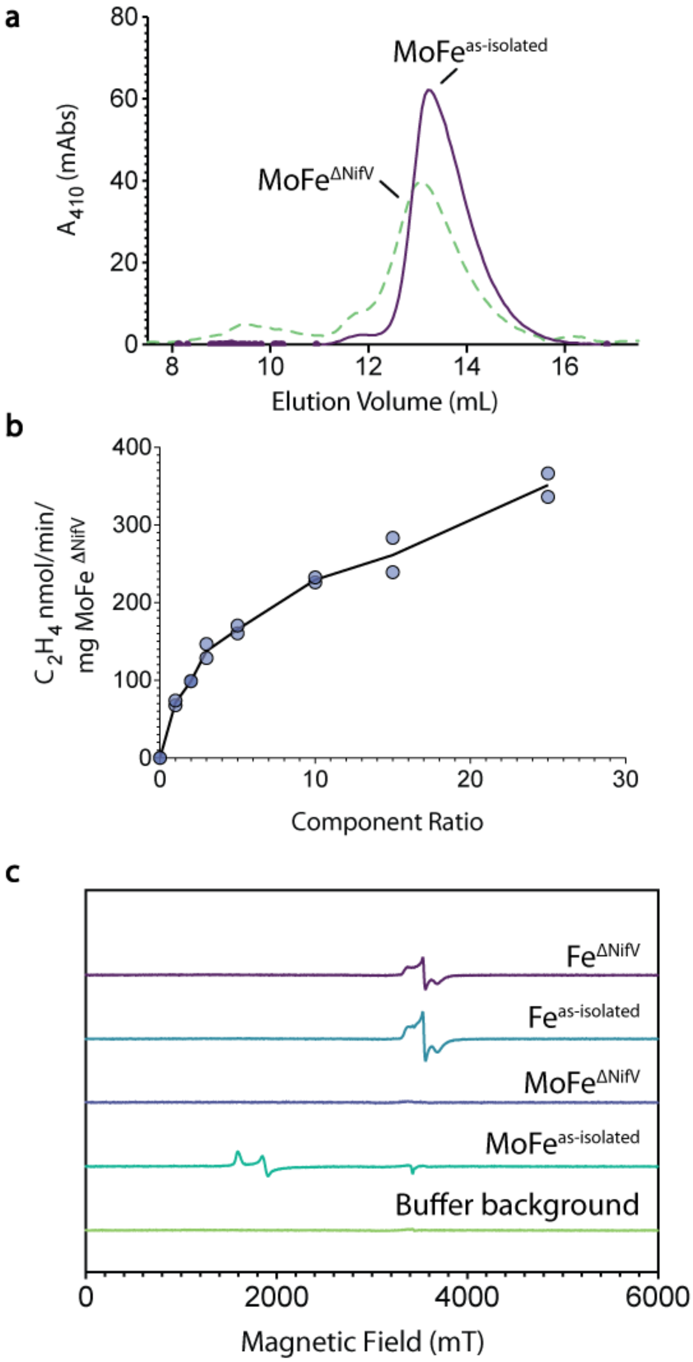

Supplementary Figure 9: MoFe$^{\Delta NifV}$ exhibits altered biochemical properties. a, Separation of MoFe$^{As\text{-}isolated}$ (solid line, purple) and MoFe$^{\Delta NifV}$ (dashed line, green) by size exclusion chromatography monitoring the elution of the metalloproteins by absorbance at 410 nm. **b,** Activity assay total nanomoles of ethylene (C_2H_4) produced as a property of component ratio (n=2 reactions, blue circles). **c,** Electron paramagnetic resonance spectra of the MoFe$^{\Delta NifV}$ and Fe $^{\Delta NifV}$ isolated from the Δ *nifV A. vinelandii*. Source data are provided as a Source Data file. Abbreviations: ATP, Adenosine triphosphate; Fe$^{As\text{-}isolated}$, purified Fe-protein; Fe$^{\Delta NifV}$, Fe-protein purified from Δ*nifV A. vinelandii* ; C_2H_4, ethylene; mAbs, milli-absorbance units; mT, milliTesla.

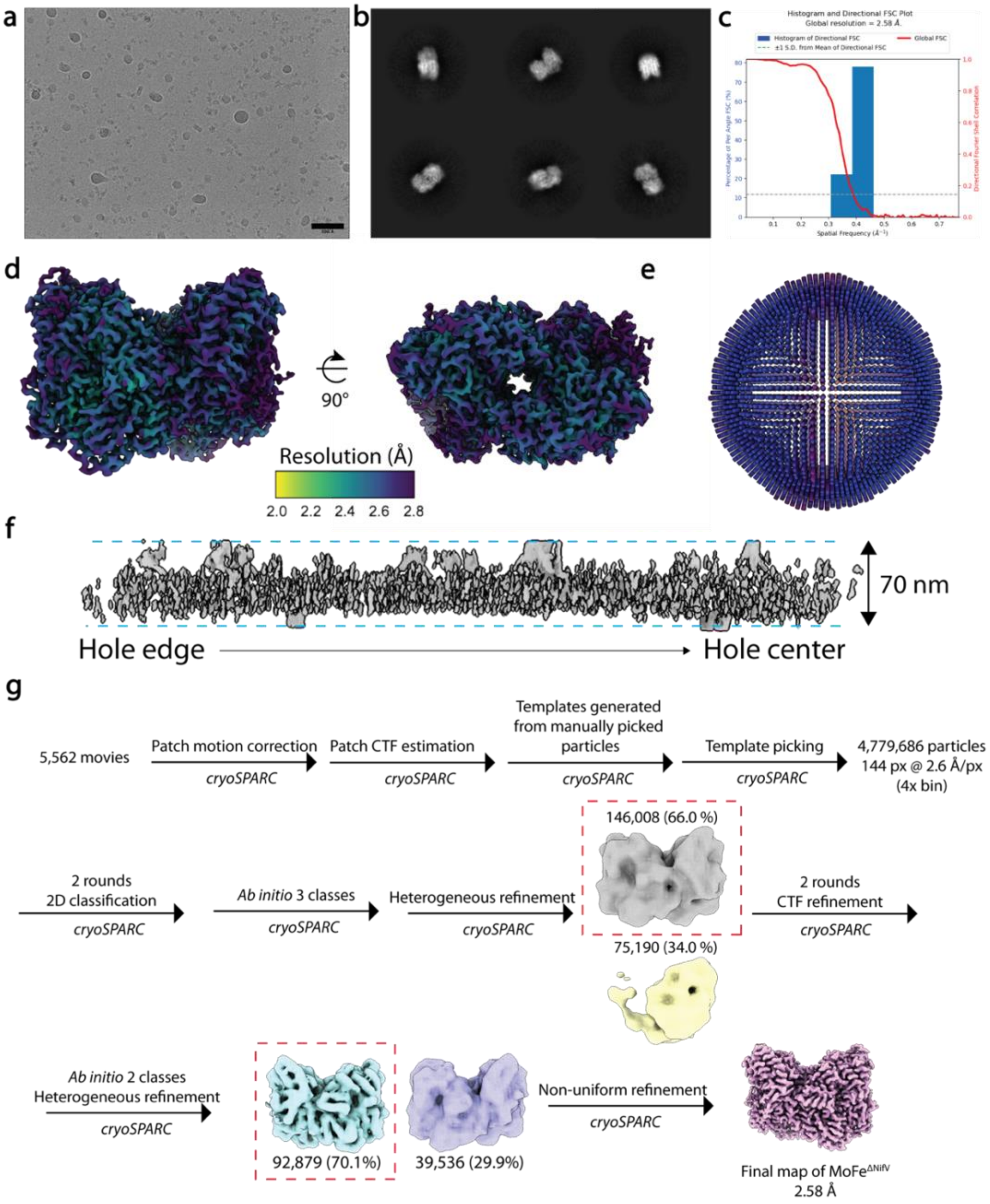

Supplementary Figure 10: Single particle cryoEM characterization of MoFe$^{\Delta\ NifV}$. a, Representative micrograph of MoFe$^{\Delta NifV}$ particles from a dataset of 5,562 micrographs. **b,** Representative 2D classes of MoFe$^{\Delta NifV}$ particles **c,** Half map FSC curve for reconstructed volume. **d**, Cryo-EM map color-coded according to local resolution estimates. **e**, Euler angle distributions from reconstructed single particle cryo-EM map (Red, overrepresented views). **f,** Volumetric representation of tomogram showing particle distribution in ice. The solid gray features correspond to the reconstructed volumes of the hole edge. **g**, CryoEM data processing workflow. Abbreviations: Å, Ångstroms; nm, nanometers; FSC, Fourier Shell Correlation; px, pixel; CTF, contrast transfer function.

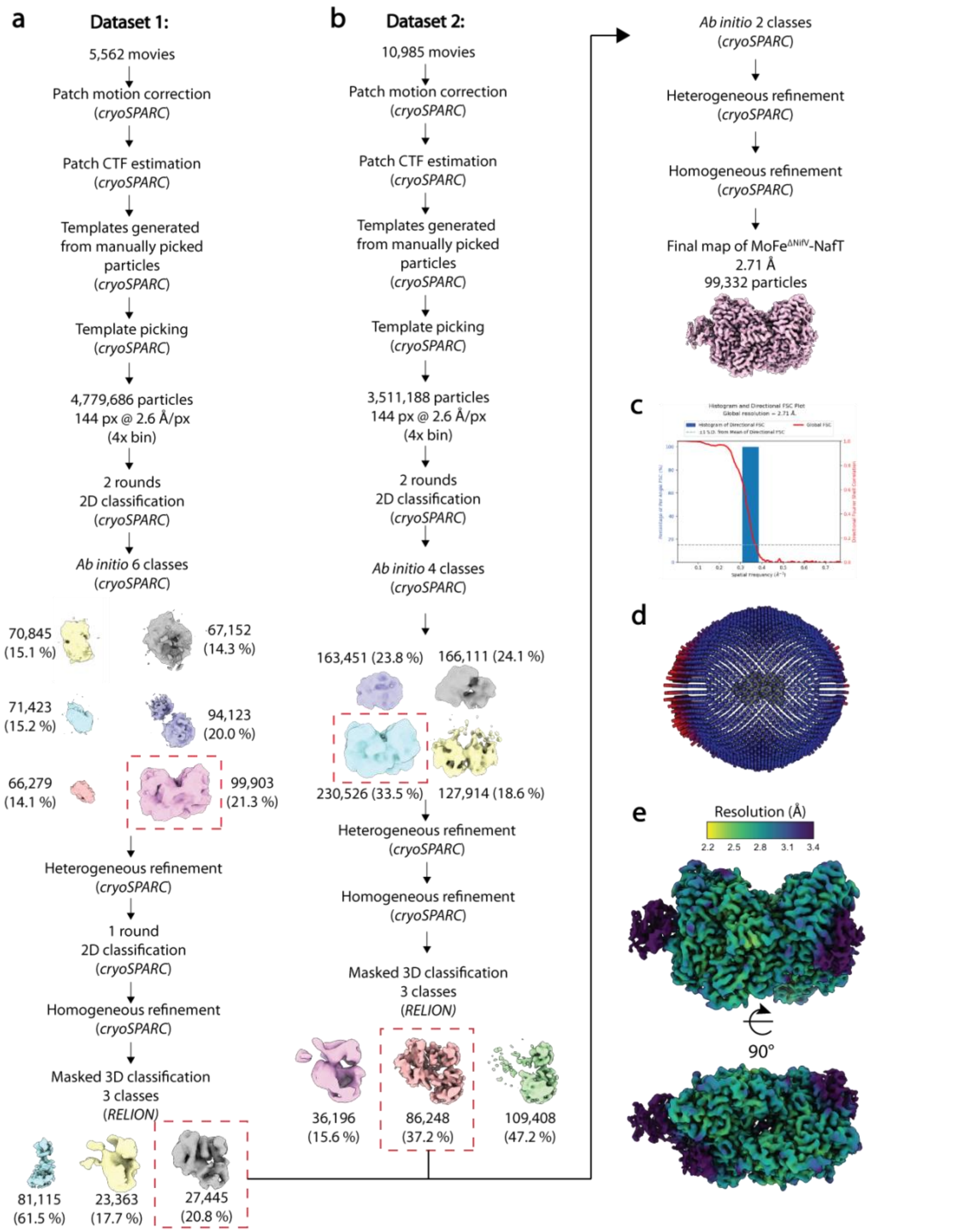

Supplementary Figure 11: Single particle cryoEM characterization of MoFe$^{\Delta NifV}$-NafT. a-b, CryoEM data processing workflow for subclassification of MoFe$^{\Delta NifV}$-NafT particles. Dataset 1 is the same dataset as that in Supplementary Figure 9, reprocessed for extraction of the complex particles. Dataset 2 is additional data of the same sample in total constituting 16,547 micrographs. **c,** Half map FSC curve for reconstructed volume. **d**, Euler angle distributions from reconstructed single particle cryo-EM map (Red, overrepresented views). **e**, Cryo-EM map color-coded according to local resolution estimates. Abbreviations: Å, Ångstroms; nm, nanometers; FSC, Fourier Shell Correlation; px, pixel; CTF, contrast transfer function.
