# Supplementary material for: Structural consequences of turnover-induced homocitrate loss in nitrogenase
Source: Nat Commun. 2023 Feb 25;14:1091. doi: 10.1038/s41467-023-36636-4 (PMC9968304; doi:10.1038/s41467-023-36636-4)
Supplement: Supplementary file 3 — Description of Additional Supplementary Files [file 41467_2023_36636_MOESM3_ESM.pdf]

Description of Additional Supplementary Files

File name: Supplementary Data 1

Description: Proteomic hits from mass spectrometry experiment.

File name: Supplementary Movie 1

Description: Three dimensional variability analysis component 0 of the $MoFe^{Alkaline\text{-}inactivated}$ cryoEM map with density corresponding to α-subunits color-coded in blue and β-subunits in yellow.

File name: Supplementary Movie 2

Description: A morph between the $MoFe^{Alkaline\text{-}inactivated}$ and $MoFe^{As\text{-}isolated}$ structures illustrates a similar movement, with shifts apparent in αIII domain helices Pro α302 – Lys α315, Glu α318 – Arg α345, and Lys α426 – Met α434, and β-strand Lys α349 – Ile α355.
