# Supplementary material for: Structural consequences of turnover-induced homocitrate loss in nitrogenase
Source: Nat Commun. 2023 Feb 25;14:1091. doi: 10.1038/s41467-023-36636-4 (PMC9968304; doi:10.1038/s41467-023-36636-4)
Supplement: Supplementary file 7 — Reporting Summary [file 41467_2023_36636_MOESM7_ESM.pdf]

nature portfolio

Corresponding author(s): Douglas C. Rees
Rebeccah A. Warmack

Last updated by author(s): 01/31/2023

Reporting Summary

Nature Portfolio wishes to improve the reproducibility of the work that we publish. This form provides structure for consistency and transparency in reporting. For further information on Nature Portfolio policies, see our Editorial Policies and the Editorial Policy Checklist.

Statistics

For all statistical analyses, confirm that the following items are present in the figure legend, table legend, main text, or Methods section.

n/a	Confirmed	
☐	☒	The exact sample size (*n*) for each experimental group/condition, given as a discrete number and unit of measurement
☐	☒	A statement on whether measurements were taken from distinct samples or whether the same sample was measured repeatedly
☐	☒	The statistical test(s) used AND whether they are one- or two-sided<br>*Only common tests should be described solely by name; describe more complex techniques in the Methods section.*
☐	☒	A description of all covariates tested
☐	☒	A description of any assumptions or corrections, such as tests of normality and adjustment for multiple comparisons
☐	☒	A full description of the statistical parameters including central tendency (e.g. means) or other basic estimates (e.g. regression coefficient) AND variation (e.g. standard deviation) or associated estimates of uncertainty (e.g. confidence intervals)
☐	☒	For null hypothesis testing, the test statistic (e.g. *F*, *t*, *r*) with confidence intervals, effect sizes, degrees of freedom and *P* value noted<br>*Give P values as exact values whenever suitable.*
☐	☒	For Bayesian analysis, information on the choice of priors and Markov chain Monte Carlo settings
☐	☒	For hierarchical and complex designs, identification of the appropriate level for tests and full reporting of outcomes
☒	☐	Estimates of effect sizes (e.g. Cohen's *d*, Pearson's *r*), indicating how they were calculated

Our web collection on statistics for biologists contains articles on many of the points above.

Software and code

Policy information about availability of computer code

Data collection	Bruker Win-EPR software; Chromeleon 7.2.10 software; Agilent MassHunter software; SerialEM
Data analysis	Proteome Discoverer SEQUEST (version 2.5, Thermo Scientific); GraphPad Prism version 9.0.0; Pymol version 2.4.2; ChimeraX 1.4; cryoSPARC 3.3; RELION 4.0; Phenix 1.20.1-4487; RefMac Servalcat CCPEM 1.6.0; Coot 0.9.8.4; Warp; IMOD version 4.11.21; IsoNet 0.2

For manuscripts utilizing custom algorithms or software that are central to the research but not yet described in published literature, software must be made available to editors and reviewers. We strongly encourage code deposition in a community repository (e.g. GitHub). See the Nature Portfolio guidelines for submitting code & software for further information.

Data

Policy information about availability of data

All manuscripts must include a data availability statement. This statement should provide the following information, where applicable:

- Accession codes, unique identifiers, or web links for publicly available datasets
- A description of any restrictions on data availability
- For clinical datasets or third party data, please ensure that the statement adheres to our policy

The single particle cryoEM maps and models generated in this study have been deposited into the PDB and EMDB for release upon publication. Reconstructed maps and refined models been deposited with the following PDB and EMDB codes: 8CRS [https://doi.org/10.2210/pdb8crs/pdb], EMD-26957 [https://www.ebi.ac.uk/emdb/entry/EMD-26957] (MoFeAs-isolated); PDB 8DBX [https://doi.org/10.2210/pdb8dbx/pdb], EMD-27316 [https://www.ebi.ac.uk/emdb/entry/EMD-27316]

(MoFeOxidized); PDB 8ENL [https://doi.org/10.2210/pdb8enl/pdb], EMD-28272 [https://www.ebi.ac.uk/emdb/entry/EMD-28272] (MoFeAlkaline-inactivated); PDB 8ENM [https://doi.org/10.2210/pdb8enm/pdb], EMD-28273 [https://www.ebi.ac.uk/emdb/entry/EMD-28273] (MoFeAlkaline); PDB 8ENN [https://doi.org/10.2210/pdb8enn/pdb], EMD-28274 [https://www.ebi.ac.uk/emdb/entry/EMD-28274] (MoFeΔNifV); PDB 8ENO [https://doi.org/10.2210/pdb8eno/pdb], EMD-28275 [https://www.ebi.ac.uk/emdb/entry/EMD-28275] (MoFeΔNifV-NafT). The Uniprot all-reviewed A. vinelandii database was used for analysis of mass spectrometry data. All other data are available from the corresponding authors upon reasonable request. Source data are provided with this paper.

Human research participants

Policy information about studies involving human research participants and Sex and Gender in Research.

Reporting on sex and gender	N/A
Population characteristics	N/A
Recruitment	N/A
Ethics oversight	N/A

Note that full information on the approval of the study protocol must also be provided in the manuscript.

Field-specific reporting

Please select the one below that is the best fit for your research. If you are not sure, read the appropriate sections before making your selection.

☒ Life sciences ☐ Behavioural & social sciences ☐ Ecological, evolutionary & environmental sciences

For a reference copy of the document with all sections, see nature.com/documents/nr-reporting-summary-flat.pdf

Life sciences study design

All studies must disclose on these points even when the disclosure is negative.

Sample size	Sample size of cryoEM datasets were determined by access to microscope time and a desire to achieve the highest resolution possible. Details of total micrographs collected are provided in Supplemental Table 1. Sample size of biochemical assays, including enzymatic reactions and MS experiments, were chosen on the basis of established laboratory practices and requirements for statistical analysis.
Data exclusions	During cryoEM data processing, picked particles were excluded during data processing in cryoSPARC on the basis of standard criteria for selection of images with the highest resolution content. Details of particles selected are provided in Supplemental Table 1. In Supp. Fig. 4, one replicate of the 0 hour time point for the minus ATP reaction was excluded due to a mispipetted reaction. Data values for replicates in biochemical experiments are provided in the Source Data file.
Replication	Biochemical assays, including enzymatic reactions and MS experiments, were performed in duplicates and triplicates under controlled conditions which allowed for assessment of reproducibility. Data can be viewed within the Source Data file. Nearly identical reconstructions from independent control cryoEM datasets, including the MoFe as-isolated, MoFe alkaline, MoFe oxidized, and MoFe high dose maps, support the reproducibility of the samples and the processing.
Randomization	For calculations of cryoEM map resolution, the cryoSPARC software randomly splits selected particles into two groups for gold standard Fourier Shell Correlation. Biochemical assays were not randomized as they do not include clinical trials or experiments with live organisms.
Blinding	Blinding was not performed in this study. Steps were taken to reduce possible interpretation bias including the collection and quantification by standardized procedures on dedicated equipment.

Reporting for specific materials, systems and methods

We require information from authors about some types of materials, experimental systems and methods used in many studies. Here, indicate whether each material, system or method listed is relevant to your study. If you are not sure if a list item applies to your research, read the appropriate section before selecting a response.

Materials & experimental systems

n/a	Involved in the study
☒	☐ Antibodies
☒	☐ Eukaryotic cell lines
☒	☐ Palaeontology and archaeology
☒	☐ Animals and other organisms
☒	☐ Clinical data
☒	☐ Dual use research of concern

Methods

n/a	Involved in the study
☒	☐ ChIP-seq
☒	☐ Flow cytometry
☒	☐ MRI-based neuroimaging
